# Systems Genetic Analyses Highlight a TGFβ-FOXO3 Dependent Striatal Astrocyte Network Conserved across Species and Associated with Stress, Sleep, and Huntington’s Disease

**DOI:** 10.1371/journal.pgen.1006137

**Published:** 2016-07-08

**Authors:** Joseph R. Scarpa, Peng Jiang, Bojan Losic, Ben Readhead, Vance D. Gao, Joel T. Dudley, Martha H. Vitaterna, Fred W. Turek, Andrew Kasarskis

**Affiliations:** 1 Icahn Institute for Genomics and Multiscale Biology, Department of Genetics and Genomic Sciences, Icahn School of Medicine at Mount Sinai, New York, New York, United States of America; 2 Center for Sleep and Circadian Biology, Department of Neurobiology, Northwestern University, Evanston, Illinois, United States of America; Merck Research Laboratories, UNITED STATES

## Abstract

Recent systems-based analyses have demonstrated that sleep and stress traits emerge from shared genetic and transcriptional networks, and clinical work has elucidated the emergence of sleep dysfunction and stress susceptibility as early symptoms of Huntington's disease. Understanding the biological bases of these early non-motor symptoms may reveal therapeutic targets that prevent disease onset or slow disease progression, but the molecular mechanisms underlying this complex clinical presentation remain largely unknown. In the present work, we specifically examine the relationship between these psychiatric traits and Huntington's disease (HD) by identifying striatal transcriptional networks shared by HD, stress, and sleep phenotypes. First, we utilize a systems-based approach to examine a large publicly available human transcriptomic dataset for HD (GSE3790 from GEO) in a novel way. We use weighted gene coexpression network analysis and differential connectivity analyses to identify transcriptional networks dysregulated in HD, and we use an unbiased ranking scheme that leverages both gene- and network-level information to identify a novel astrocyte-specific network as most relevant to HD caudate. We validate this result in an independent HD cohort. Next, we computationally predict FOXO3 as a regulator of this network, and use multiple publicly available in vitro and in vivo experimental datasets to validate that this astrocyte HD network is downstream of a signaling pathway important in adult neurogenesis (TGFβ-FOXO3). We also map this HD-relevant caudate subnetwork to striatal transcriptional networks in a large (n = 100) chronically stressed (B6xA/J)F2 mouse population that has been extensively phenotyped (328 stress- and sleep-related measurements), and we show that this striatal astrocyte network is correlated to sleep and stress traits, many of which are known to be altered in HD cohorts. We identify causal regulators of this network through Bayesian network analysis, and we highlight their relevance to motor, mood, and sleep traits through multiple in silico approaches, including an examination of their protein binding partners. Finally, we show that these causal regulators may be therapeutically viable for HD because their downstream network was partially modulated by deep brain stimulation of the subthalamic nucleus, a medical intervention thought to confer some therapeutic benefit to HD patients. In conclusion, we show that an astrocyte transcriptional network is primarily associated to HD in the caudate and provide evidence for its relationship to molecular mechanisms of neural stem cell homeostasis. Furthermore, we present a unified systems-based framework for identifying gene networks that are associated with complex non-motor traits that manifest in the earliest phases of HD. By analyzing and integrating multiple independent datasets, we identify a point of molecular convergence between sleep, stress, and HD that reflects their phenotypic comorbidity and reveals a molecular pathway involved in HD progression.

## Author Summary

Huntington’s disease is a complex neurodegenerative disorder caused by CAG expansion in the huntingtin gene. Huntington’s disease leads to abnormal involuntary movements in patients, but recent studies have shown that many non-motor psychiatric and sleep changes also manifest in the earliest phases of the disease, often before gross changes in movement. This study identifies gene networks that are affected by Huntington’s disease and associated with these non-motor traits, revealing biological pathways and the cell-types likely involved in the earliest stages of Huntington’s disease.

## Introduction

Huntington’s disease (HD) is a progressive and fatal neurodegenerative disorder caused by abnormal expansion of the CAG repeat in the huntingtin gene (HTT). Mutant huntingtin protein causes variable morphological pathology and differential gene expression throughout the brain, with the striatum exhibiting the earliest and most severe effects[1]. Consequently, patients suffering from HD most notably develop motor abnormalities, including chorea and dystonia. However, HD patients also develop significant non-motor symptoms, including depression, anxiety, and sleep disturbance, that are often associated with stress and typically precede significant neuronal loss and the onset of motor dysfunction by many years[2–9]. Understanding the biological bases of these early non-motor symptoms may reveal therapeutic targets that prevent disease onset or slow disease progression[4,10], but the molecular mechanisms underlying this complex clinical presentation remain largely unknown.

**Fig 1:**
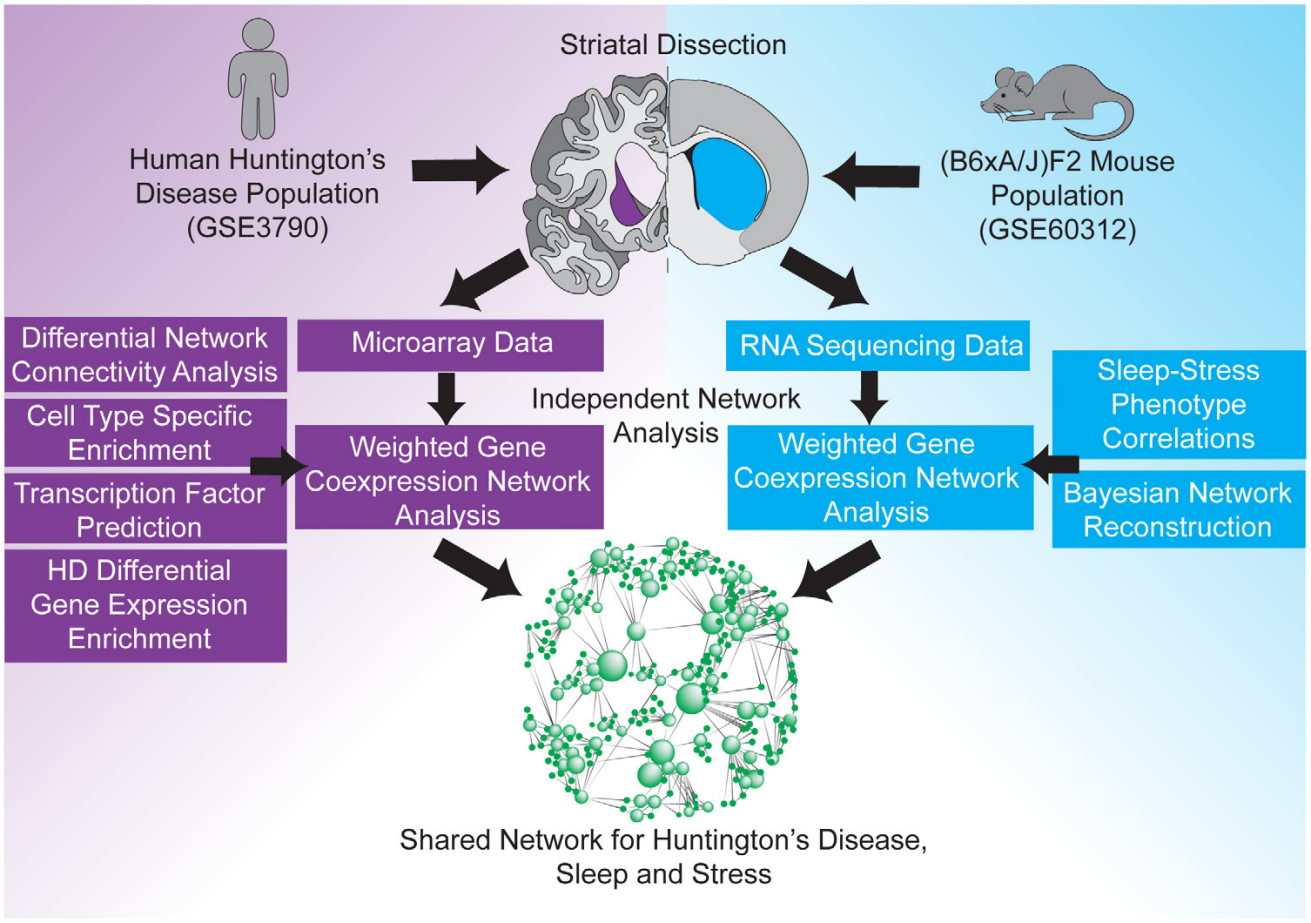
Outline of the integrative analysis framework for relating sleep and stress (non-motor) traits to specific HD-associated transcriptional networks in the striatum. HD-relevant gene networks were identified in a human HD cohort (GSE3790) and unbiasedly mapped to gene networks independently identified in a (B6xA/J)F2 mouse population associated with stress susceptibility and sleep. This strategy revealed a shared gene network associated with sleep, stress, and HD.

There is rapidly accumulating evidence that many genetic and molecular factors contribute to complex phenotypes[11,12], and recent systems-based analyses suggest that sleep and stress traits, in particular, emerge from shared genetic and transcriptional networks[10]. These results have led to the hypothesis that common networks shared between sleep, stress, and neurodegenerative diseases may elucidate novel pathological mechanisms and reveal therapeutic targets[10]. In particular, since stress-related psychiatric and sleep disturbances precede motor symptoms and severe neuronal loss in HD, a systems-level analysis that integrates HD-relevant gene networks with non-pathological stress and sleep gene networks may reveal convergent network domains where HTT proteotoxicity impinges on normal functions of stress response and sleep. In the present work, we tested this hypothesis explicitly by investigating common striatal transcriptional networks underlying HD, stress, and sleep phenotypes (Fig 1).

To perform this analysis, we primarily integrated two different datasets: 1) a public microarray dataset (GSE3790 from GEO) from a human HD cohort and age- and sex-matched controls; and 2) genetic, phenotypic, and RNA-sequencing data from a large (N = 100) chronically stressed (B6xA/J)F2 mouse population that has been extensively phenotyped (328 stress- and sleep-related measurements). Previous work has described systems-based pathologies involved in the onset and progression of HD[13–16], and some groups have explored a variety of approaches for combining different types of HD-relevant data[17,18]. In this work, we integrate gene and network-level analyses to identify transcriptional networks whose expression and connectivity are dysregulated in HD and evaluate which HD-associated networks are associated with stress susceptibility and sleep disruption. We revealed significant alterations to molecular networks in the caudate, cortex, and cerebellum of a human HD cohort that were not previously appreciated by differential expression alone. We found that an astrocyte network is most relevant to HD pathology in the caudate and showed that this astrocyte HD network is downstream of a signaling pathway important in adult neurogenesis (TGFβ-FOXO3). We found that this HD-relevant astrocyte network is conserved in the striatum of a (B6xA/J)F2 mouse population and is correlated to several sleep and stress measures implicated in HD. Lastly, we identified the candidate causal regulators of this astrocyte network through Bayesian network analysis and showed that their downstream nodes are significantly modulated by deep brain stimulation, a medical intervention thought to confer some therapeutic benefit to HD patients[19,20]. By analyzing and integrating multiple independent datasets, we identified a point of molecular convergence between sleep, stress, and HD that reflects their phenotypic comorbidity and supports a fundamental mechanism of neuropathogenesis.

## Results

### Gene network remodeling in the caudate, cerebellum, and frontal cortex of a human HD cohort

**Fig 2:**
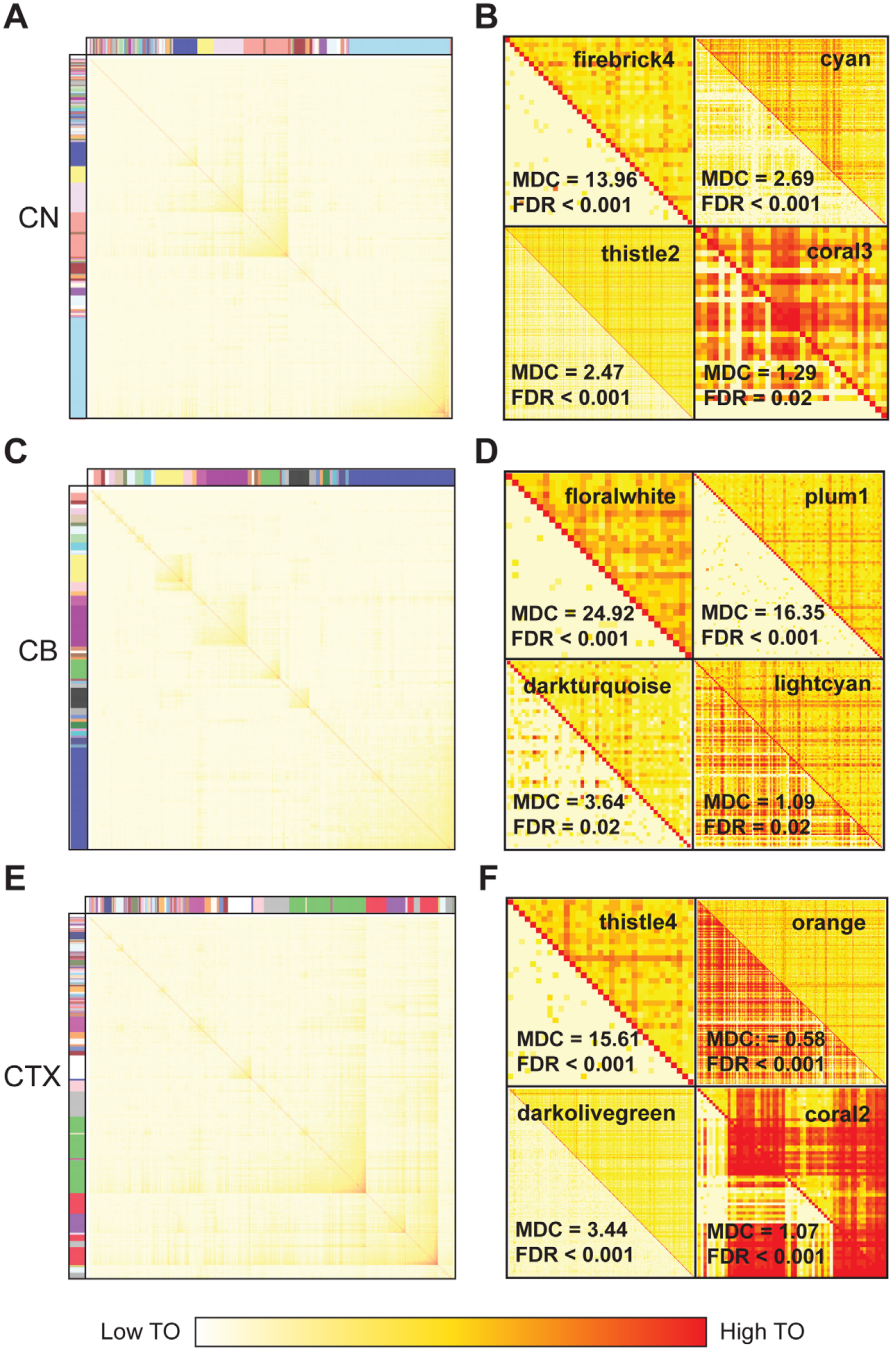
Coexpression networks in the CN, CB, and CTX of a human HD cohort. Topological overlap (TO) matrix plot depicts gene coexpression networks in (A) CN, (C) CB, and (E) CTX. (B,D,F) A comparison of TO matrices in cases (top right triangle) versus controls (bottom left triangle) for selected modules in each brain region. High TO (greater coexpression) is colored red, while low TO is colored white. Module differential connectivity (MDC) and FDR values are depicted for each module. Differential connectivity was considered significant by conservative thresholds (MDC > 2.0 or MDC < 0.5, FDR < 0.001). Three differentially connected modules and one conserved module are depicted for each brain region.

To identify HD-relevant molecular networks, we examined publicly available human microarray gene expression data (GSE3790) from 203 brain samples. The HD-gene-positive cases include 39 caudate nucleus (CN) samples, 38 cerebellar (CB) samples, and 37 frontal cortex (CTX) samples, and the age- and sex-matched controls include 28 CN, 32 CB, and 29 CTX samples. Using weighted gene coexpression network analysis[21], we constructed transcriptional networks of the CN, CB, and CTX from the HD cohort, and we identified 85, 36, and 96 modules in the three brain regions, respectively (Fig 2A, 2C and 2E). Each module represents a group of coexpressed genes, named using an arbitrarily assigned color and determined to be robust through resampling methods (S1 Fig). As a resource, we report network-based statistics and module membership assignments for each module in S1–S3 Tables.

**Fig 3:**
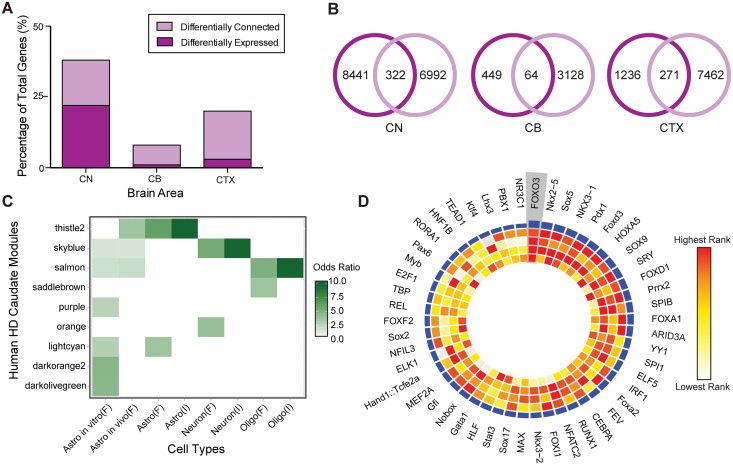
Network-specific pathology and functional characterization of CN Thistle2 module. (A,B) Differential connectivity analysis reveals network-level alterations (light purple) that were not observed by previous differential expression analysis in the same cohort^1^ (dark purple). (B) Venn diagrams depict the number of genes identified by differential connectivity (light purple) and differential expression analyses (dark purple), as well as their overlap. (C) CN modules showing enrichment for previously published cell-type specific gene signatures identified by FACS (F) and in situ hybridization (I) experiments. Fisher’s exact test odds ratios are plotted only for modules with P < 0.05, two-sided, Bonferroni corrected. (D) Circos plot depicting FOXO3 as the top TF associated with Thistle2 in CN; rings are numbered 1 (outermost) to 5 (innermost). TF binding site enrichment scores are depicted in rings 2, 3, and 4 (Z score, Fisher’s score, and Composite Rank, respectively). Ring 5 depicts the differential expression profile of each TF in HD (-log10(P)). Blue histogram height (ring 1) reflects the cumulative scores of each TF based upon rings 2–5, with taller heights depicts greater relevance to Thistle2.

To identify modules most relevant to HD, we compared modules between the pathological cohort and a cohort of age- and sex-matched controls. For each brain region, we calculated modular differential connectivity (MDC), which measures changes in connectivity in HD-associated modules with respect to their module counterpart in the control cohort[11]. We identified differentially connected molecular networks in all three brain regions (Fig 2B, 2D and 2F) and confirmed these results using an independent methodology that measures module preservation[22] (S2 Fig). We note that differential connectivity signatures capture more transcriptional information between cases and controls than was previously appreciated by differential expression alone[1] (Fig 3A and 3B). This is best exemplified in the cerebellum, a region thought to be largely spared in HD. While very few cerebellar genes are differentially expressed between cases and controls, differential connectivity analyses reveal that HD strongly alters gene networks in the cerebellum, affecting 6-fold more genes than was estimated by differential expression experiments. These significant changes to cerebellar gene networks are consistent with more recent evidence for macro- and microstructural damage to the cerebellum in HD and its association to both motor and non-motor symptoms[23].

Since differential connectivity reveals novel pathological information, we investigated if network-level analysis supported the common notion that HD primarily affects the caudate. Hodges et al. (2006) argues that the number of differentially expressed genes in HD parallels morphological pathology (caudate>cortex>cerebellum)[1], and we note that this pattern is also reflected in the number of genes in differentially connected networks, as expected. However, we note that pathological changes to molecular networks are far more equally dispersed across brain regions than differential expression analyses have revealed, supporting the notion that HD is a multi-focal and systemic brain disease[13,24](Fig 3A). For example, previous work with the same human cohort reported that the number of differentially expressed genes in the cerebellum are approximately 5% of the number of differentially expressed genes in the caudate[1], but this difference is far less pronounced when comparing differentially connected genes (40%). Taken together, these results further support the prominence of caudate pathology in HD. However, they also reveal that the HTT mutation causes more significant and widespread effects across multiple brain regions than previously appreciated by differential expression analysis alone.

### An astrocyte module is most relevant to HD caudate pathology

Since the caudate network is most significantly affected in HD, we focused our downstream analysis on this brain region. We reasoned that modules most relevant to HD would exhibit both gene- and network-level dysregulation, so we ranked modules by their MDC score and their enrichment for differentially expressed genes in HD (S4 Table). We calculated module enrichment for two published HD differential expression signatures—calculated in this cohort (GSE3790) and a replication cohort (GSE26927 from GEO)–in order to ensure robustness. Several modules were either differentially connected or enriched with differential expressed genes. A neuron-specific module (Skyblue), which includes D1 and D2 dopamine receptors, and a module involved in apoptotic regulation (Lightcyan) both showed significant gene-level dysregulation, but only small changes in network connectivity. On the other hand, a cytoskeletal module (Blue) and an antigen processing and presentation module (White) were differentially connected in HD, but not enriched robustly for differentially expressed genes. Our unbiased ranking revealed that Thistle2 was the module most relevant to HD, since it was the only module that was both strongly differentially connected (MDC = 2.47, FDR < 0.001) and overrepresented with HD differential expression signatures from both datasets (GSE26927: *P* = 5.7 x 10^−23^; GSE3790: *P* = 7.1 x 10^−19^; Bonferroni corrected, Fisher’s exact test), particularly genes upregulated in HD (GSE26927: *P* = 3.2 x 10^−31^; GSE3790: *P* = 1.5 x 10^−52^).

Next, we investigated whether Thistle2 was overrepresented with cell-type specific gene signatures derived by fluorescence-activated cell sorting (FACS)[25]. We noted enrichment for an astrocyte gene expression signature (*P* = 1.1 x 10^−82^), which was specific to *in vivo* astrocytes (*P* = 3.0 x 10^−40^) and not cultured astrocytes (*P* > 0.05). Thistle2 was not overrepresented with either neuronal (*P* > 0.05) or oligodendrocyte (*P* > 0.05) gene signatures. We also noted Thistle2 was enriched with an independent astrocyte gene signature derived from *in situ* hybridization[26](*P* = 4.7 x 10^−10^). Lastly, we found that this module was overrepresented with an astrocyte-specific proteome signature as well [27] (*P* = 2.6 x 10^−84^). These analyses support the conclusion that Thistle2 is astrocyte specific using multiple independently derived cell-type specific signatures across two orthogonal data types. Additional analyses confirmed that this module enrichment was not correlated with pathological grade, suggesting it is unlikely that this network simply reflects astrocytosis (S3 Fig). Furthermore, to show that this HD-relevant astrocyte network was reproducible, we examined microarray data from an independent human cohort (GSE26927). These analyses revealed that this astrocyte module captures highly robust gene coexpression patterns that show both significant gene and network dysregulation in HD (S4 Fig). Taken together, this evidence suggests that the transcriptional module most relevant to HD pathology is astrocyte specific (Fig 3C).

### TGFβ-FOXO3 signaling regulates the HD astrocyte module

Since genes in the Thistle2 module are coexpressed, we reasoned that their common regulatory control could be driven a by shared transcription factor (TF). We identified 36 TFs predicted to regulate the astrocyte module and calculated a composite rank statistic that prioritizes TFs by their binding site enrichment score and differential expression profile in HD (S5 Table). FOXO3, an important regulator of adult neural stem cell homeostasis, was the highest-ranking transcription factor (Fig 3D). To validate that FOXO3 modulates the astrocyte module, we examined three independent datasets (GSE60137, GSE13347, and GSE18326 from GEO), in which FOXO3 was manipulated with different experimental approaches (shRNA in C2C12 cells, siRNA in C1E-ER-GATA1 cells, and FoxO3-/- murine brain, respectively). The astrocyte module was overrepresented with each of the three FOXO3-dependent differential expression profiles, confirming that FOXO3 robustly regulates the astrocyte module (GSE60137: *P* = 9.0 x 10^−7^; GSE13347: *P* = 4.3 x 10^−39^; GSE18326: *P* = 3.5 x 10^−12^).

Lastly, we investigated if FOXO3 modulation of Thistle2 is downstream of any common signaling pathways. We found that the TGFβ pathway was the most significant upstream regulator for both the Thistle2 module and its 36 predicted TFs (S5A Fig). Further, we found that FOXO3 is itself regulated by the TGFβ pathway and note that the subset of Thistle2 genes with FOXO3 binding sites also are strongly modulated by TGFβ signaling (*P* = 7.33 x 10^−8^) (S5B Fig). Together, these results implicate TGFβ-FOXO3 signaling as an important regulator of HD-associated transcriptional coexpression in the caudate.

### The HD-relevant astrocyte network is associated with sleep and sleep traits in a (B6xA/J)F2 mouse population

**Fig 4:**
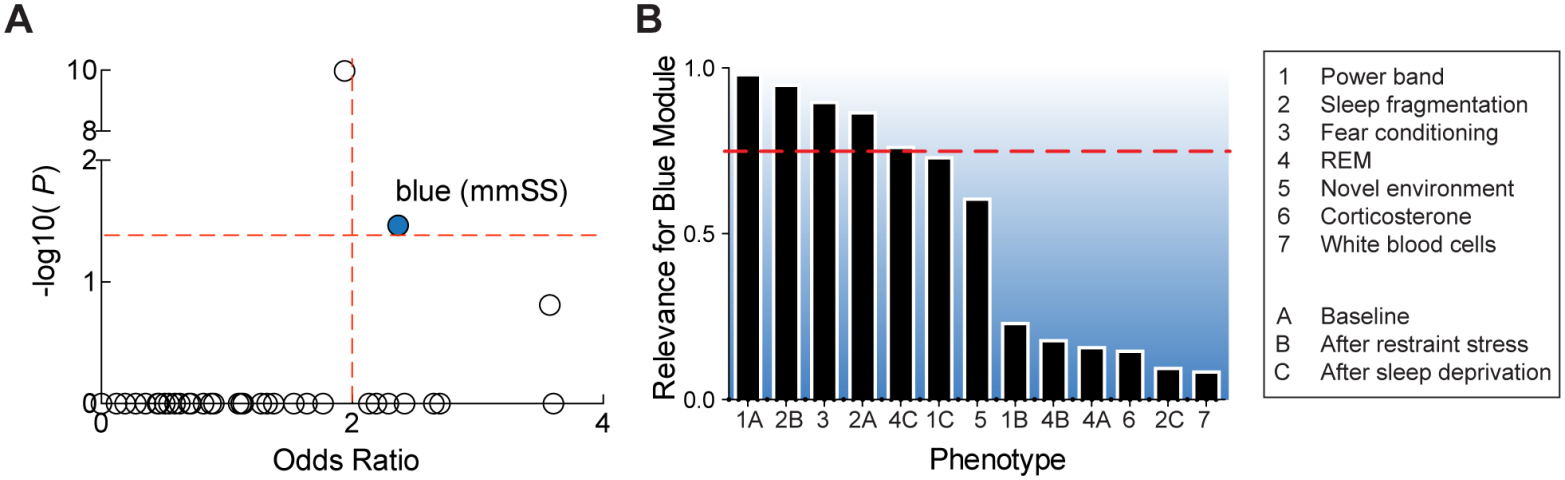
Conservation of the HD-associated Thistle2 module in mmSS cohort. (A) Scatterplot of enrichment results for Thistle2-hsHD, depicting its conservation in the Blue module of the (B6xA/J)F2 mouse cohort. Significance thresholds (red lines): Odds Ratio = 2 and P = 0.05, two-sided, Bonferroni corrected. (B) Barplots showing module-trait associations between the Blue module and several sleep and stress traits measured in the mouse cohort. Module relevance to phenotype categories are based upon significant module-trait correlations. Module-trait associations above the horizontal red line are the top ranked relationships (belonging to the top quartile).

To determine if HD-relevant networks are associated with sleep and stress traits, we investigated if any HD transcriptional networks in the caudate also were coexpressed in the striatum of a chronically stressed (B6xA/J)F2 mouse population(GSE60312 from GEO) that has been extensively phenotyped (328 stress- and sleep-related measurements)[10]. We unbiasedly mapped modules from the *H*. *sapiens* Huntington’s disease cohort (hsHD) to the modules previously calculated from RNA-seq data (N = 100) in the *M*. *musculus* sleep/stress cohort (mmSS). This analysis revealed that 18% of hsHD modules were also coexpressed in the mmSS cohort and that the Thistle2-hsHD module mapped specifically to the Blue-mmSS module (Fig 4A). Further analyses confirm that genes in Thistle2-hsHD are highly expressed and robustly measured across the human and mouse experiments[28] (S6 Fig).

To determine if the Blue-mmSS module was relevant to sleep and stress traits, we investigated if its module eigengene (1^st^ principal component of the module) was significantly correlated with any of the 328 sleep and stress traits measured in the (B6xA/J)F2 population[10]. Blue-mmSS is strongly associated with fear conditioning, sleep fragmentation after restraint stress, and baseline power band measures (Fig 4B). Notably, the human corollaries of many of these mouse traits are dysregulated in HD cohorts[2,3,29]. Overall, these analyses reveal that the HD-relevant astrocyte network is associated to non-motor (sleep and stress) traits and provide a strategy for identifying molecular pathways likely associated with non-motor symptoms.

These data suggest that Thistle2-hsHD may be relevant to early HD pathogenesis because of its associations with non-motor phenotypes. In order to corroborate this evidence, we investigated if Thistle2-hsHD gene expression is dependent upon CAG length. CAG repeat length is inversely correlated with HD onset age[30], but weakly associated with disease progression[31]. Therefore, CAG length-dependent gene expression can point to the early phases of HD molecular pathogenesis[32]. A recent comprehensive study has identified robust CAG length-dependent networks in mouse striatum[16], so we compared these networks to our HD-relevant networks. This analysis revealed that Thistle2 was most strongly overrepresented with a network whose expression was shown to be inversely correlated with CAG length (“M11”, *P* = 2.9 x 10^−8^). Previous work also demonstrated that this CAG length-dependent network (“M11”) is conserved in BACHD-ΔN17, R6/2, and HdhQ150 mice, suggesting this module is robust across mouse models of HD. Our analysis shows that a bottom-up (CAG length-dependence) and top-down (phenotype) approach to studying early phases of HD pathogenesis converge onto the Thistle2-hsHD astrocyte network.

### Candidate causal regulators of the sleep and stress network are upstream of HD-relevant nodes

**Fig 5:**
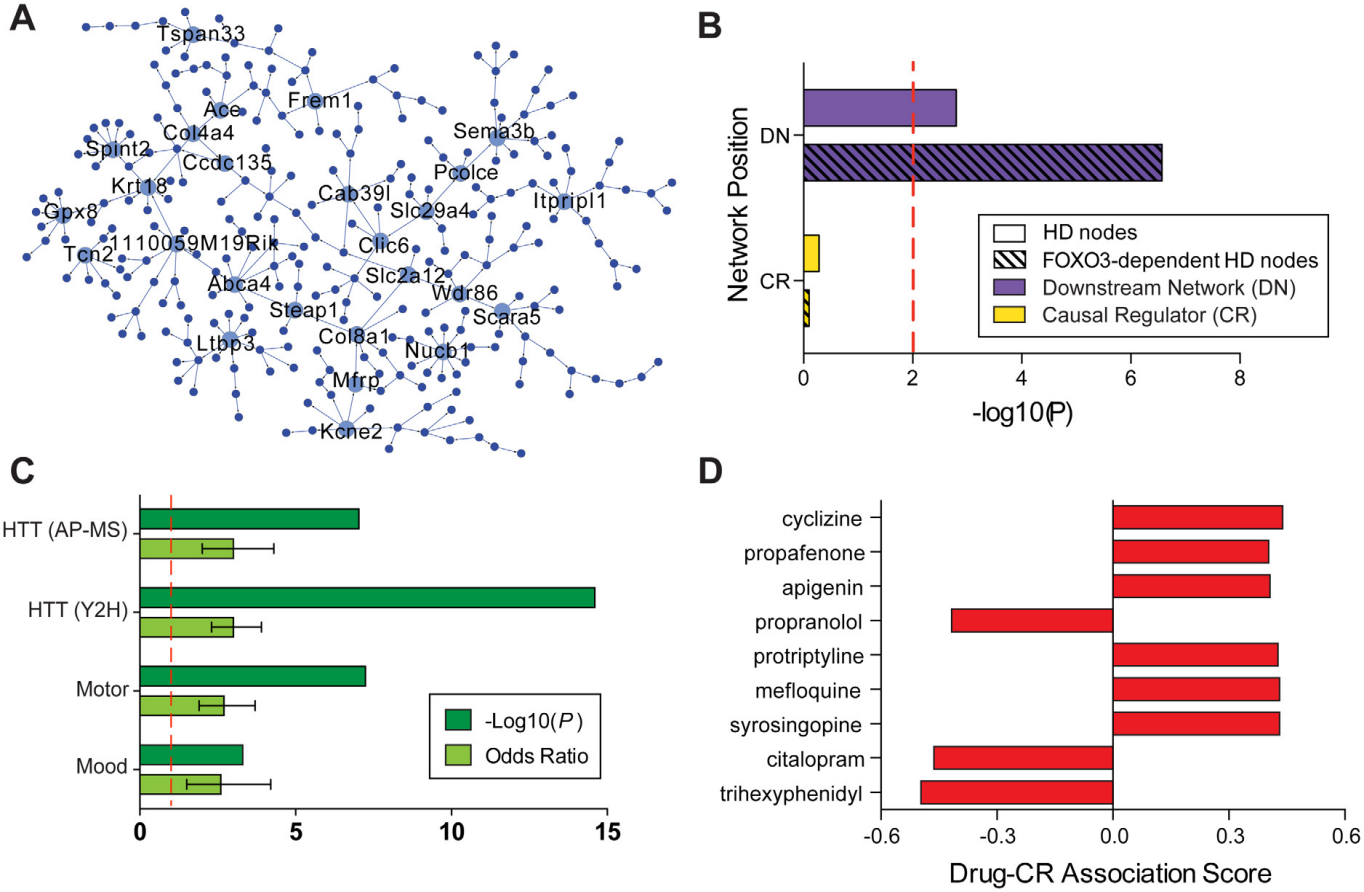
Candidate causal regulators of the Blue-mmSS module are upstream of HD-relevant nodes. (A) Bayesian network reconstruction of the Blue-mmSS reveals 26 CCRs (large labeled nodes). (B) Genes in Thistle2-hsHD, especially those with FOXO3 binding sites, are significantly overrepresented in the Blue-mmSS downstream network. Significance threshold (red line): P = 0.01, two-sided. (C) Enrichment of the causal regulator PIN for huntingtin protein binding partners identified by affinity purification-mass spectrometry (AP-MS) and yeast two-hybrid (Y2H) methods, and for genes necessary for voluntary movement and affective behavior. Significance thresholds (red line): P = 0.01, two-sided; Odds Ratio (95% Confidence Interval) = 2. (D) Drugs that concordantly upregulate (Drug-Causal Regulator Association Score > 0) and downregulate (Drug-Causal Reulgator Association Score < 0) Blue-mmSS CCRs. All drugs shown have Benjamini-Hochberg adjusted P < 0.05.

To identify the candidate causal regulators (CCRs) of the Blue-mmSS module, we used an integrative genomics approach that leveraged both genetic and gene expression data from 100 (B6xA/J)F2 mouse samples to calculate causal probabilistic relationships between genes [11,33–36]. In previous work, we identified genes whose expression was dependent on genetic variability (*cis*-eQTLs) in order to construct a Bayesian network (with *cis*-eQTLs priors) of all gene-gene relationships[10]. In this analysis, we specifically investigated the Blue-mmSS module and calculated its CCRs (nodes with a significant number of downstream partners). These CCRs drive the expression of the Blue-mmSS module, and therefore represent its most important members. We identified 26 Blue-mmSS CCRs, which include some genes that have been implicated previously in HD-associated pathology (Fig 5A). For example, *Nucb1* encodes a protein that inhibits amyloid fibril formation often observed in HD[37], and *Gpx8* encodes a glutathione peroxidase, an enzyme recently shown to be neuroprotective in HD animal models[1,38].

Next, we investigated if the CCRs of Blue-mmSS are themselves dysregulated in HD. Four CCRs (*Tcn2*, *Ace*, *Spint2*, and *Ltbp3)* are differentially expressed in the human HD cohort (GSE3790). *Ltbp3* is especially interesting since the LTBP family regulates the localization, activation, and bioavailability TGFβ[2–6,39–41], the signaling pathway that we identified as most relevant to Thistle2-hsHD in the human HD caudate. However, we note that the 26 CCRs are not enriched for differentially expressed HD genes (*P* = 0.8), while their downstream nodes are (*P* = 1.6 x 10^−3^). This relationship is further supported when examining the members of Blue-mmSS that are also coexpressed in Thistle2-hsHD. Only one Blue-mmSS CCR, *Tspan33*, is a member of the human HD Thistle2 module, while the downstream network is overrepresented for the Thistle2-hsHD module (*P* = 2.9 x 10^−6^), and more specifically for Thistle2-hsHD genes with FOXO3 binding sites (*P* = 2.7 x 10^−7^) (Fig 5B). As a whole, this evidence suggests that CCRs relevant to sleep and stress control the expression of the HD-relevant transcriptional network.

### Protein interaction network of causal regulators is associated with HTT-toxicity and motor and non-motor traits

To further support the claim that CCRs of the sleep and stress mouse network are relevant to HD, we investigated their protein interaction network (PIN). We constructed their PIN by querying first-degree protein-protein interactions previously identified through experimental and computational methods and considered only interactions between proteins that are expressed in the caudate (S6 Table). We found that the PIN was overrepresented with proteins known to cause abnormal voluntary movement (*P* = 5.7 x 10^−8^) and abnormal emotional and affective behavior (*P* = 5.3 x 10^−4^), suggesting that the CCRs strongly interact with proteins that are necessary for motor and non-motor traits relevant to HD (Fig 5C). We then investigated whether these CCRs may be directly affected by HTT-toxicity. Notably, their PIN is overrepresented with HTT-interacting proteins, identified by a yeast two-hybrid screen[42] (*P* = 2.7 x 10^−15^). We confirmed this finding with a second independent dataset that identified *in vivo* HTT-complexed proteins from a mouse model of HD by affinity purification-mass spectrometry[14] (*P* = 6.4 x 10^−7^). Overall, these results provide strong orthogonal evidence that the Blue-mmSS CCRs are relevant for both motor and non-motor phenotypes associated with HD and associated with HTT proteotoxicity.

### Drugs associated with motor and non-motor traits modulate astrocyte network CCRs

To examine if modulating CCRs is associated with motor and non-motor phenotypes, we determined which bioactive small molecules coherently affect our CCRs using transcriptional profiles in Connectivity Map. We reasoned that molecules affecting psychiatric or neurological traits may work in part by modulating candidate causal nodes in the Blue-mmSS subnetwork. This analysis revealed a number of molecules that concordantly up- or down-regulate these CCRs (*P* < 0.05, Benjamini-Hochberg corrected, Kolmogorov-Smirnov). Molecules that significantly downregulate these CCRs include drugs which ameliorate motor symptoms, depression, and anxiety. On the other hand, molecules that upregulate these candidate causal nodes include drugs which can induce dystonia, motor restlessness, extrapyramidal symptoms, jitteriness, and insomnia (Fig 5D). For example, CCRs are downregulated by citalopram (*P* = 0.0097) and trihexyphenidyl (*P* = 0.0027), treatments for depression/anxiety and Parkinson’s disease, respectively. On the other hand, these CCRs are upregulated by cyclizine (*P* = 0.0166), which has been reported to induce chorea[43–45], mefloquine (*P* = 0.0203), which can induce or exacerbate a variety of neuropychiatric and sleep phenotypes[46,47], and protriptyline(*P* = 0.0216), whose side effects include anxiety, insomnia, and decreased motor coordination. These results provide further orthogonal evidence associating these CCRs with the emergence of sleep, stress, and motor symptoms. All scores and statistics reflecting compound-causal regulator associations are provided in S8 Table.

### Deep brain stimulation of the subthalamic nucleus modulates common striatal network of HD, sleep, and stress

Deep brain stimulation (DBS) is used as a therapeutic tool in several neuropsychiatric disorders, and recent reports suggest that DBS targeting the globus pallidus internus and the subthalamic nucleus (STN) may ameliorate motor and non-motor symptoms in HD patients[19,20]. Therefore, we tested the therapeutic viability of the astrocyte network by investigating whether it is affected by striatal transcriptional changes induced by DBS to the STN. Previous work has characterized the striatal molecular signature induced by STN-DBS in the rat[48], and we find that this striatal DBS signature is overrepresented in both Thistle2-hsHD (*P* = 1.5x 10^−2^) and Blue-mmSS (*P* = 6.5 x 10^−5^). We note that the DBS signature exclusively affects downstream nodes of the Blue-mmSS subnetwork, suggesting the potential therapeutic benefits of upstream modulation. *Slc4a2* is directly modulated by DBS and is immediately downstream of *Ltbp3*, discussed above as a CCR of our network and an important modulator of TGFβ signaling. While *Slc4a2* knockout causes motor deficits[49], its upregulation is associated motor symptom resolution after STN-DBS[48]. Though the relationship between *Slc4a2* and TGFβ pathway has been proposed in other systems[50], our network analysis reveals that this mechanism may contribute to the therapeutic benefits of DBS. In sum, STN-DBS modulates a striatal astrocyte network relevant to both motor and non-motor symptoms of HD, suggesting that regulating this network may confer therapeutic benefit.

## Discussion

Our work provides a systems-based approach for identifying and prioritizing networks and pathways relevant to HD, and our analysis has revealed many novel insights into the molecular mechanisms underlying HD pathogenesis. Previous systems analyses have identified gene networks in post-mortem HD brain correlated with pathological grade[15], while other work has investigated neural stem cells differentiated from HD-patient derived iPSCs and revealed gene networks whose module eigengene expression distinguishes neural stem cells with the CAG expansion from neural stem cells genetically corrected with homologous recombination-based gene targeting methods[51]. More recent work has used allelic series knock-in mice to identify CAG length-dependent gene networks in order to study early HD pathogenesis[16]. In our work, we leverage a multi-species population approach to study early HD pathogenesis that integrates genetic, transcriptomic, proteomic, and behavioral data across human and mouse cohorts to identify HD-relevant networks associated with non-motor traits prevalent in early HD. We provide evidence that an astrocyte module is the caudate network whose connectivity and expression is most altered in HD. We confirmed its coexpression in an independent cohort, associated it with FOXO3-TGFβ signaling, showed it was CAG length-dependent, and correlated it with many sleep and stress phenotypes in a (B6xA/J)F2 mouse population, including several traits that have been previously identified in human HD populations. Lastly, we show that CCRs of this sleep and stress network control the HD-associated nodes and are strong therapeutic candidates.

Most functional and molecular work on HD has focused on medium spiny neurons, but our analyses instead suggest a primary role for astrocytes in HD striatal pathology. Our results also suggest that the HD-relevant astrocyte network is insufficiently explained by astrocytosis, since module enrichment for astrocyte-specific genes was not correlated with pathological grade. Given this evidence, we argue that this astrocyte network is critical to HD pathogenesis, a conclusion also supported by data from the prefrontal cortex in multiple HD cohorts[24,52]. Other evidence suggests that astrocyte-specific expression of the mutant huntingtin protein is sufficient to cause the striatal neuron degeneration[53] and age-dependent neurological symptoms[54] observed in HD, and both the altered neuronal excitability[55] and chronic inflammation[56] in HD may be the result of primary astrocyte dysfunction. Our integrative network analyses, accompanied by these functional data, support the hypothesis that astrocytes are a primary contributor to HD pathogenesis. However, alternative interpretations may be considered when interpreting these data. For instance, the nature of post-mortem brain samples make it difficult completely rule out astrocytosis as a partial contributor to this HD-relevant network. Also, it is possible that astrocyte network dysregulation in early HD may be secondary to increased oligodendrocyte density[57]. Further work is required to understand how astrocytes affect neuropathological and phenotypic features of early HD.

We integrated our network analysis with experimental knockdown data and highlighted that this astrocyte network is downstream of TGFβ-FOXO3 signaling. Previous work has studied the role of the FOXO homologue (*daf-16*) in a *Caenorhabditis elegans* model of early mutant htt toxicity in which expanded polyQs cause a defective touch response but minimal cell death[58–60]. These studies demonstrate an important role for FOXO in improving touch response in 128Q nematodes. Other evidence in immortalized striatal cell lines demonstrates that Sirt1 requires Foxo3a to make cells with mutant HTT resilient to serum withdrawal, complementing *C*. *elegans* evidence for the protective effects of FOXO3a[61]. While these data suggest that FOXO3 is neuroprotective and repressed in HD, other evidence from R6/2 mice and human post-mortem caudate argues that FOXO3 overexpression is associated with HD partly due to an overactive autofeedback loop[62]. These seemingly conflicting data suggest that HD-relevant mechanisms are dynamic, multifactorial, and perhaps not always well-conserved between animal models and human disease. Our analytical strategy was agnostic to these neuroprotection hypotheses and did not intend to resolve these problems. Instead, our data-driven analyses suggest that FOXO3 regulates a human striatal astrocyte gene network that is CAG length-dependent and associated with non-motor phenotypes relevant to early HD. These data implicate a novel pathway through which FOXO3 can influence HD pathogenesis. We note that previous experiments focused on FOXO3-dependent effects on neurons and often did not directly assay astrocytes, and this gap may also partially explain conflicting conclusions drawn by previous literature. It is intriguing that FOXO3 may exert its protective or degenerative effects through specific cell-types, or through different cell-types at different stages of HD pathogenesis, but further work needs to be done to disentangle these complex cellular phenomena.

Dysregulation of neurotrophic and growth factors in HD are also well documented. Previous work has identified a HD-associated gene network in iPSC-derived neural stem cells that is associated with TGFβ signaling[51]. It is especially notable that two different approaches to studying early HD pathogenesis implicate the TGFβ pathway since previous molecular work on non-motor HD symptoms tend to focus on the TrkB pathway[2,63]. The importance of TGFβ signaling in early HD pathogenesis is further supported by work demonstrating that valproic acid and lithium, both of which have been shown to improve mood in HD patient[63–65], can affect TGFβ signaling[66,67]. Furthermore, TGFβ has been noted as an important regulator of FOXO transcription factors in a number of conditions and tissues[68,69], and our analysis ties this mechanism to an astrocyte gene network in HD caudate. It is also important to note that several signaling pathways converge onto FOXO transcription factors[70–72] and function through FOXO3 in HD pathogenesis[60]. These findings emphasize the importance of studying system-level gene interactions in order to understand and disentangle the complex and multifactorial mechanisms associated with HD. Lastly, adult generated neurons are depleted in the striatum of HD patients[73], and both FOXO3 and TGFβ play fundamental roles in regulating and maintaining adult neural stem cell populations[74–77]. Furthermore, dysregulation of neural differentiation networks has been associated with glial pathogenesis in the human prefrontal cortex of both Alzheimer’s disease and Huntington’s disease cohorts[52]. Our results link these molecular mechanisms of adult neural stem cell maintenance to astrocyte pathology in the caudate, which is consistent with previous work showing that *FoxO3* dysregulation disrupts neural stem cell homeostasis by specifically skewing the population toward astrocyte lineages[76,78]. Overall, our analyses suggest astrocyte pathophysiology and neural stem cell depletion in HD may emerge from a single pathological mechanism.

We mapped this astrocyte network to independently derived striatal networks from chronically stressed (B6xA/J)F2 mice and showed that its expression correlated with stress and sleep phenotypes. Previous studies have demonstrated that astrocytes can modulate cortical slow oscillation, sleep amount, and sleep drive[79–81] and can contribute to sleep-loss induced deficits[82]. Our work presents strong evidence that alterations in mood and sleep traits are associated with the HD astrocyte network in the striatum. Each of these phenotypes has been previously identified in human HD patients, but our systems-based analyses associate these seemingly disparate phenotypes to a HD-relevant transcriptional network in the caudate. We also showed that the expression of this astrocyte network is dependent upon CAG length, further suggesting its role in early HD pathogenesis. However, it is important to note that previous work found insufficient evidence to corroborate the genotype-expression correlation of these genes in the human cohort (GSE3790). This difference may be explained partly by limitations in power across a diverse range of CAG length in the human cohort. Differences in cell population changes between the mouse and human cohorts also may have confounded this approach for these specific astrocyte genes. Nevertheless, we emphasize that our multi-species population approach and the allelic series knock-in mouse models both implicate this astrocyte network in early HD pathogenesis. The convergence of these approaches was further supported by showing that the primary module described by Langfelder et al (“M2”) was overrepresented with previously identified F2 networks, Turquoise-mmSS and Mediumpurpl2-mmSS[10]. These F2 networks partly motivated the present study since they were strongly associated with sleep, stress susceptibility, and neuropsychiatric disease and suggested that HD-relevant pathways are direct modulators of sleep and stress phenotypes. One of these networks was driven by *Htt* (Mediumpurple2-mmSS) and a second network (Turquoise-mmSS) includes *Rrm2b* and *Ncald* among its most connected genes (hubs), two genes near a locus associated with earlier clinical onset of HD[83]. In sum, these analyses strongly suggest that two independent strategies for studying early HD pathogenesis implicate similar pathways and integrating these data revealed specific sleep and stress traits correlated with CAG length-dependent striatal networks that are altered in human HD.

Further network analyses revealed that sleep and stress CCRs control the expression of the HD-relevant nodes and are affected by HTT-toxicity. The organization within this shared network reflects the emergence of stress- and sleep-related phenotypes in the early phases of HD and the eventual manifestation of significant motor symptoms later in the disease. It also suggests upstream modulation of this sleep and stress subnetwork may be therapeutically efficacious by delaying the progression of HD. These results support the notion that psychiatric symptoms are a primary clinical presentation of HD and suggest that targeting the molecular mechanisms of non-motor symptoms may significantly improve both psychiatric and motor function in HD patients, as has been done with antipsychotics like risperidone[84].

Notably, we show that this network shared between sleep, stress, and HD is affected by deep brain stimulation of the subthalamic nucleus, a medical intervention that ameliorates both the motor and non-motor symptoms in some HD patients[20]. Functional data support our network analysis, implicating astrocytes in the therapeutic mechanism of action of DBS[85,86]. However, we note that DBS does not modulate any CCRs of this astrocyte network, which may explain the limited and short-term benefits of this therapy in HD. Robust modulation of this network may lead to more efficacious treatment, and our network analysis can serve as a resource for evaluating therapeutic options *in silico* and prioritizing future *in vivo* experiments.

In these analyses, we have shown that sleep, stress, and HD share an astrocyte network affected by signaling pathways of adult neurogenesis, and our systems-based approach provides a framework for considering psychiatric symptoms as the primary manifestation of neurodegenerative disease. It is important to note that not all HD patients experience the same non-motor symptoms, but this astrocyte network is a striatal pathway through which HD can impinge upon sleep and stress traits in genetically or environmentally susceptible individuals. By integrating molecular networks from comorbid psychiatric and neurological conditions, we can identify novel points for therapeutic intervention, prioritize mechanisms of neurodegenerative pathogenesis, and perhaps even capture molecular dynamics of disease progression, which are difficult to measure directly in human brain.

Though this work makes significant steps towards systematically studying the common molecular basis of motor and non-motor features of HD, we acknowledge that our analysis focuses on only one of many points of intersection worthy of further exploration. We also acknowledge that further *in vivo* investigation of Thistle2-Blue network is essential for completely understanding the functional and morphological consequences of each CCR and the best points of therapeutic intervention. This may include an expanded investigation of transcription factor binding and upstream pathway regulation. We validated FOXO3, but 35 other transcription factors also are predicted to control the Thistle2 module, including several that are differentially expressed in the HD caudate. Some predicted transcription factors, like HOXA5 and FOXF2, have also been implicated in HD pathogenesis in the prefrontal cortex[24]. Lastly, our results indicate that HD causes significant changes to transcriptional networks across the caudate, frontal cortex, and cerebellum. While this work emphasizes caudate-specific pathophysiology, it also provides evidence that HD-relevant transcriptional networks in other brains regions are significantly associated with pathogenesis and require proper investigation, a molecular feature not previously captured by differential expression. Specifically, three cerebellar networks (Blue, Royalblue, Midnightblue) exhibit both gene- and network-level changes in HD and would be strong candidates for HD-relevant functional studies in the cerebellum (S9 Table). These modules are associated with protein folding and oxidative phosphorylation functional pathways, and one cerebellar module (Royalblue) is even overrepresented with the Huntington’s disease Kyoto Encyclopedia of Genes and Genomes (KEGG) pathway, suggesting that HD does not spare the cerebellum. This conclusion is well supported by more recent pathological studies that indicate profound changes in brain regions outside the striatum (like brainstem nuclei and cerebellum), which may play significant roles in motor and non-motor symptom manifestation, especially in the early phases of the disease[23,87–89]. Therefore, we believe a comprehensive understanding of HD requires further investigation of both the mechanisms underlying its comorbid psychiatric and neurological features and the effects of pathology on networks across multiple brain regions.

## Materials and Methods

### HD expression data and correction

Hodges et al. (2006) studied differential gene expression across three brain regions in a cohort of 44 HD-gene-positive cases and 36 age- and sex-matched controls using Affymetrix microarray HG-U133A and U133B[1]. HD cases had between 42–46 CAG repeats while unaffected controls ranged from 17–21. The pathological range is considered greater than 35 repeats. Neuropathological staging of the HD cohort ranges from 0–4, and the majority of HD cases were graded as Grade 1 or 2. Three cases (graded as either 0 or 1) were considered presymptomatic. We downloaded the author-normalized (MAS5) expression data and associated covariate table from GEO (GSE3790). The dataset consists of 203 samples, with 67 cerebellar samples, 70 caudate samples, and 66 frontal cortex samples. For each brain region, we further adjusted the expression data for age and sex by fitting a robust linear regression model and taking the residuals as the corrected expression levels. Lastly, we used surrogate variable analysis to account for unknown and unmeasured covariates[90]. The same protocol was used to prepare the expression data from the validation cohort (GSE26927).

### Identifying the transcriptional landscape for HD

We generated coexpression modules from the corrected expression data in the HD cohort (GSE3790), using weighted gene coexpression analysis[21]. We identified linear relationships between gene expression using pairwise Pearson correlation and defined the “scale-free” adjacency matrix of the gene expression graph by raising the Pearson correlation matrix to a positive power, β[21]. Nearest-neighbor links were accounted for by quadratically transforming the adjacency matrix into topological overlap matrix (TOM), which depicts biological interactions with high fidelity[91]. Coexpression modules were then defined using a hierarchical clustering method that implements the Dynamic Tree Cut algorithm[92]. Modules were arbitrarily assigned colors, with the grey module identifying genes failing to segregate into a particular module. We assessed the robustness of each module by repeatedly splitting the data into training and test sets and calculating a module preservation score between the resulting networks[22]. Module association to pathological grade was quantified by relating each module’s 1^st^ principal component to pathological grade with a Kruskal-Wallis test, and module-grade associations were expressed as its –log10(*P*). Fisher’s exact test was used to assess module enrichment for cell-type specific and HD differential expression signatures, and Bonferroni corrected *p*-values are reported for all Fisher’s exact tests. We used cell-type specific signatures derived from FACS and *in situ* hybridization experiments, and we tested module enrichment for HD differential expression profiles derived from this cohort (GSE3790) and a second cohort (GSE26927) in order to ensure robustness and reproducibility. Module conservation was tested for all HD modules with greater than 50 module members. Fischer’s exact test was used to determine module conservation between the HD cohort and the (B6xA/J)F2 cohort, for which a module was considered conserved with Bonferroni corrected *p*-value < 0.05 (accounting for all module-module comparisons) and Odds Ratio > 2. Module conservation was similarly assessed between the HD cohort and the allelic series knock-in cohort. When necessary, homology information provided by Ensembl was used for human-mouse conversion. In the case of many-to-many homology, all possible pairs of human and mouse genes were kept.

Recent work has demonstrated that the RNA-seq and microarray estimates of gene expression are most divergent for lowly expressed genes, while genes with higher expression are robustly estimated across assays[28]. To ensure that our coexpression graph overlap for the astrocyte module (Thistle2) was not biased by lowly expressed genes that were poorly estimated across assays, we examined median gene expression in all modules calculated in the human HD cohort. We note that genes with low expression were difficult to distinguish from noise (background hybridization), so unsupervised clustering tended to assign them to “grey” (no module). Consequently, the median expression of genes in grey was systematically lower than genes in modules with a color assignment, as expected (Kolmogorov Smirnov *P* < 2.2 x 10^−16^) (S6A and S6B Fig). In general, this suggests that genes assigned to coexpression modules are not significantly affected by any systematic bias attributable to the microarray. Further, we directly compared Thistle2 to all other modules (including grey). Genes in Thistle2 are among the most highly expressed (S6C Fig), suggesting that they are robustly measured within and between assays.

### Phenotypic, genetic and gene expression data in chronically stressed (B6 x A/J) F2 mice

In a previous study characterizing mouse striatal gene networks underlying multiple stress and sleep phenotypes, we described in detail a set of phenotypic, genetic and RNA-Seq gene expression data collected in a chronically stressed population of 338 male (B6×A/J) F2 mice[10]. Briefly, starting from 4–5 weeks of age, all F2 mice were subjected to a battery of stressors, including social isolation, novel exposed environments (elevated plus maze, open field, elevated zero maze), restraint, forced swimming, fear conditioning, social defeat, cold exposure, a metabolic stressor (6-hr fast and glucose tolerance test), and the sleep behavior response to sleep deprivation and restraint. Extensive behavioral and physiological measurements were taken during the chronic stress treatment. Sleep/wake behavior was recorded from each mouse using electroencephalography and electromyography. Following all stress and sleep tests, animals were euthanized by decapitation, and blood and tissue samples were collected for additional analyses. Genotypes of all animals were determined from DNA extracted from tail-tip biopsies by using the Illumina medium density single nucleotide polymorphism (SNP) panel. Gene expression profiling in the striatum of a randomly selected subset of 100 animals was performed using RNA sequencing. 100 base pair single-end sequencing reads were aligned against the Ensembl NCBIM37 mouse reference genome for gene-level expression profiling (expression data available at GSE60312). Weighted gene coexpression analysis was used to characterize the transcriptional landscape of the striatum, and 1st principal components of each module were correlated with phenotypes measured in the cohort to determine module-trait associations. The relevance of a module to a phenotype was determined by ranking the number of significant module-trait associations for each module (*P* < 0.05, FDR < 0.05).

### Calculating module differential connectivity (MDC) between HD cases and controls

As previously described[11], we calculated a MDC statistic for each module by quantifying the ratio of the average connectivity of its genes in the disease network to that of the same genes in the control network. Consequently, MDC > 1 indicates that a module gained connectivity in disease, while MDC < 1 suggests lost connectivity. In this work, we used stricter thresholds to ensure the robustness of our results (MDC >2 and MDC < 0.5). We estimated two separate false discovery rates (FDR) by randomly shuffling samples and genes of disease and control networks. Shuffling samples creates networks with random edges and shuffling genes creates networks with random nodes. We quantified the final FDR by selecting the larger estimate and used a conservative FDR threshold to assess significance (FDR < 0.001). We also confirmed that the MDC statistic revealed similar module preservation features as the medianRank statistic calculated with a separate analytical method [22]. This procedure calculates a rank-based measure that accounts for a variety of network metrics of density, connectivity, and separability, which MDC does not directly assess. MDC and medianRank statistics are highly concordant in their assessment of module preservation, as demonstrated in S2 Fig. After confirming the robustness of the MDC statistic, we integrated MDC scores and differential expression enrichment to identify modules most affected by HD on the gene and network levels. To confirm this strategy with an orthogonal method, we calculated coexpression networks from all caudate gene expression data (cases and controls in a single expression matrix) and identified modules associated with case-control status. This analysis revealed an astrocyte module strongly associated with HD, which was highly overlapping with the Thistle2 module from the original analysis, providing support for our analytical approach (S10 Table).

### Using Bayesian network reconstruction to identify network CCRs

As described in our previous work[10], we reconstructed directed acyclic graphs called Bayesian networks to identify relationships between the gene expression profiles of each gene in our network. Edges between nodes reflect the conditional probability distributions of each node given the expression of its parent node[33,93]. Using a Monte Carlo Markov Chain (MCMC) simulation, we reconstructed one thousand gene networks that fit our data, as assessed by Bayesian Information Criterion (BIC)[94]–a method conservative to overfitting. A consensus network was calculated using thresholds identified to be ideal in balancing recall rate and precision[34].

Bayesian networks do not necessarily contain causal information, since many graphical models are Markov equivalent and causally indistinguishable. By integrating genetic data in the form of expression quantitative trait loci (eQTLs), we can improve network reconstruction, as has been validated by simulation[34] and *in vivo* experimentation[33,35], and infer causality between nodes by breaking symmetrical Markov equivalent structures and improve network reconstruction.

We identified causal regulators in our directed acyclic graph by calculating the size of the h-layer neighborhood (HLN) downstream of each gene in our network[95]. Nodes whose HLN is one standard deviation greater than the mean were considered causal regulators, since they have significantly more downstream partners than the average node in the module.

### Assessing overrepresentation of transcription factor binding sites and pathway perturbations in coexpressed genes

To predict transcript factor binding, we used the oPOSSUM database[96]. Using this tool, we collected genomic DNA sequences of the Thistle2 module from the Ensembl database and identified phylogenetically conserved non-coding DNA sequences using ORCA[97]. We then used vertebrate-specific position specific scoring matrices (PSSMs) catalogued by JASPAR[98] to identify relationships between transcription factor binding sites (TFBS) and their target DNA binding motifs, restricting the search space for TFBS to phylogenetically conserved, non-coding DNA to improve specificity[99,100]. Two statistics, Z score and Fisher’s exact probability, were used to quantify TFBS enrichment, in order to account for the rate of occurrence of TFBSs and the overall proportion TFBSs in our coexpressed genes, respectively[101]. We used empirical thresholds of Z > 10 and Fisher’s < 0.01 to identify significant TFBS enrichment, which have a false positive rate of ~15%. However, we note that our effective false positive rate is significantly lower, since all of our significant targets have Z > 10 and Fisher’s *p* < 1 x 10^−10^. We ranked all transcription factors by their Z score and Fisher score, as well as by their differential expression profile in the HD cohort, to identify a prioritized list of significant transcription factors.

To identify relevant signaling pathways upstream of the Thistle2 module and its predicted TFs, we utilized 215 heterogeneous microarray experiments curated by the Signaling Pathway Enrichment using Experimental Data Sets (SPEED) database. This methodology identifies elements of the transcriptome consistently regulated by pathway perturbations across many experiments[102]. Enrichment of our gene signatures in experimentally validated downstream pathways was assessed by Fisher’s exact test. We used default criteria (z = 1%, overlap = 20%, expression = 50%) for identifying gene signatures of each pathway and accounted for multiple hypotheses testing by quantifying the false discovery rate (FDR) using the hypergeometric distribution[103]. The sensitivity and specificity of this method are 83% and 98%, respectively.

### Constructing the protein interaction network (PIN)

We constructed a PIN by integrating 13 different sources that describe known and predicted protein-protein interactions, which was compiled by HDNetDB (hdnetdb.sysbiolab.eu). These databases include data from yeast two-hybrid screens (MDC, CCSB), literature curation (HTT-HTP, Human Protein Reference Databse, BioGRID, Reactome, Database of Interating Proteins, BIND, IntAct, HomoMINT), computational text mining (COCIT), and orthology-based predictions (OrthoDB, OPHID, HomoMINT). The overwhelming majority (~90%) of edges in our constructed PIN are derived from databases curating experimentally validated protein-protein interactions, and more than half of all edges were derived from either REACTOME[104] or Human Protein Reference Database[105] (S6 Table). We assessed PIN enrichment for HTT-interacting proteins[14,42] and proteins known to cause motor and mood abnormalities with Fisher's exact test. Proteins relevant for motor and mood abnormalities (S7 Table) were curated by the Mouse Genome Informatics database and are retrievable through the mouse phenotype identifiers MP0003491 and MP0002572, respectively[106].

### Associating causal regulators with drug induced transcriptional changes

CCRs of the Blue-mmSS module were used to query Connectivity Map, a large library of drug induced transcriptional profiles[107]. We merged the 6,100 individual experiments into a single representative signature for the 1,309 unique small molecule compounds, according to the prototype-ranked list method[108]. For each unique compound, a modified Kolmogorov-Smirnov (KS) score was calculated[107], summarizing each drug’s transcriptional relationship to our Blue-mmSS CCRs and quantifying the tendency for those genes to be concordantly up- or downregulated in the presence of a given compound. Significance of individual scores was estimated by generating an empirical KS score distribution from the query network to 1,000 permuted drug signatures, and compounds with *P* < 0.05 (Benjamini-Hochberg corrected) were considered significant.

## Supporting Information

S1 Fig Module robustness calculations through resampling. Z < 2 (blue line) suggest there is weak evidence for module robustness, while Z > 10 (green line) denotes strong evidence for their reproducibility. Evidence is considered moderate when 10>Z>2. All modules identified in (A) caudate, (B) cerebellum, and (C cortex of HD-gene-positive cohort have Z quality scores > 10 (green line), suggesting they are robust, reproducible, and high quality.
(TIF)

S2 Fig Independent measures of differential connectivity. Assessing the reproducibility of the MDC statistic by comparing it to an independent methodology for assessing differential connectivity (medianRank statistic) in the (A) caudate (ρ = 0.86, *P* = 1.08 x 10^−25^), (B) cerebellum (ρ = 0.93, *P* = 6.84 x 10^−16^), and (C) frontal cortex (ρ = 0.86, *P* = 2.84 x 10^−20^).
(TIF)

S3 Fig Comparing pathological grade to astrocyte profiles in CN modules. Correlating each module’s association to pathological grade with (A) its enrichment for astrocyte gene signatures and (B) its percentage of astrocyte genes. Pathological grade is not associated to the astrocyte transcriptional profile of modules, suggesting module enrichment is not an artifact of astrocytosis.
(TIF)

S4 Fig Replication of the HD-relevant astrocyte network in an independent cohort. (A) Weighted gene coexpression analysis was performed in an independent validation cohort (N = 62) (GSE26297). In this cohort, multiple brain regions were collected from multiple neuropsychiatric diseases, including the caudate from HD patients. (B) Enrichment for HD-relevant differential expression signatures revealed that Grey60 was most relevant to HD (*P <* 0.05, two-sided, Bonferroni corrected). (C) Grey60 was also associated to HD status in the caudate (***: *P* = 3.9e-05, Kruskal-Wallis, Bonferroni corrected, df = 2–1 = 1). (d) Grey60 from our replication cohort (GSE26297) most significantly overlapped with our Thistle2 module from our original cohort (GSE3790). These results show that the caudate astrocyte network is coexpressed in an independent cohort and is most relevant to HD.
(TIF)

S5 Fig Identifying upstream pathways for FOXO3-Thistle2. (A) Enrichment of pathway perturbation signatures (Materials and Methods) with Thistle2 and its predicted TFs. Significance threshold (red line): FDR = 0.01. (B) TGFβ pathway enrichment for TFs, Thistle2, and FOXO3-dependent Thistle2. Significance threshold (red line): *P* = 0.01, FDR = 0.01, Fold enrichment = 2.
(TIF)

S6 Fig Comparing median expression between coexpression modules. (A,B) Comparison of median expression between genes that cluster in modules and those that do not (grey) (***: Kolmogorov-Smirnov *P <* 2.2 x 10^−16^). (c) Comparing median expression of genes in each module. Left dotted red line represents the top decile, and the right dotted red line represents the bottom decile. The grey module falls within the bottom decile, as expected, while Thistle2 falls within the top decile.
(TIF)

S1 Table HD caudate module membership and connectivity statistics. (XLSX)

S2 Table HD cerebellum module membership and connectivity statistics. (XLSX)

S3 Table HD frontal cortex module membership and connectivity statistics. (XLSX)

S4 Table HD caudate module ranking. Module ranks are based upon differential connectivity and differential gene expression enrichment, denoted by a "1" in columns 2 through 6.
(XLSX)

S5 Table Transcription factor binding site ranking. (XLSX)

S6 Table Causal regulator protein interaction network. (XLSX)

S7 Table Mouse Genome Informatics gene sets for abnormal vountary movement and abnormal affective behavior. (XLSX)

S8 Table Drug-causal regulator association statistics. (XLSX)

S9 Table HD cerebellum module ranking. (XLSX)

S10 Table Caudate coexpression modules associated with case/control status. This table includes module membership and connectivity statistics for modules calculated from a single gene expression matrix that includes all caudate cases and controls. It also includes module-status association P values, as well module enrichment P values for astrocyte signatures and the original Thistle2 module. All reported P values are Bonferroni corrected.
(XLSX)
