# Supplementary figures and images for: Systems Genetic Analyses Highlight a TGFβ-FOXO3 Dependent Striatal Astrocyte Network Conserved across Species and Associated with Stress, Sleep, and Huntington’s Disease
Source: PLoS Genet. 2016 Jul 8;12(7):e1006137. doi: 10.1371/journal.pgen.1006137 (PMC4938493; doi:10.1371/journal.pgen.1006137)

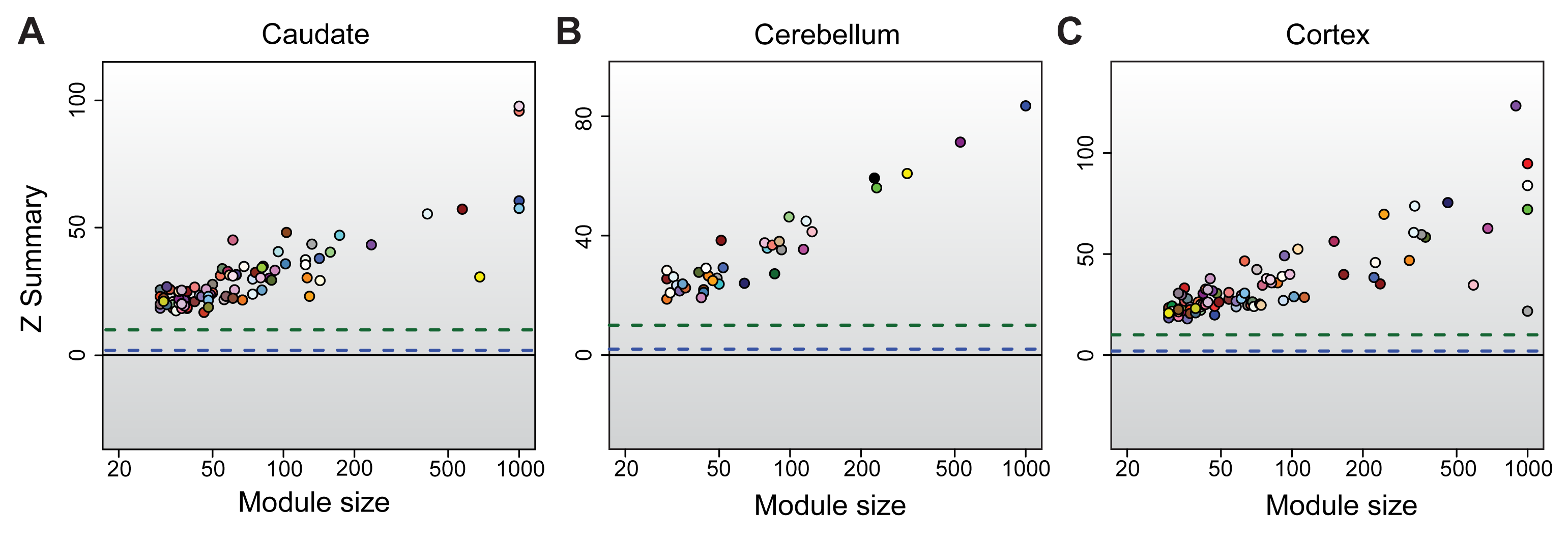

Supplement: S1 Fig — Z < 2 (blue line) suggest there is weak evidence for module robustness, while Z > 10 (green line) denotes strong evidence for their reproducibility. Evidence is considered moderate when 10>Z>2. All modules identified in (A) caudate, (B) cerebellum, and (C cortex of HD-gene-positive cohort have Z quality scores > 10 (green line), suggesting they are robust, reproducible, and high quality. (TIF) [file pgen.1006137.s001.tif]

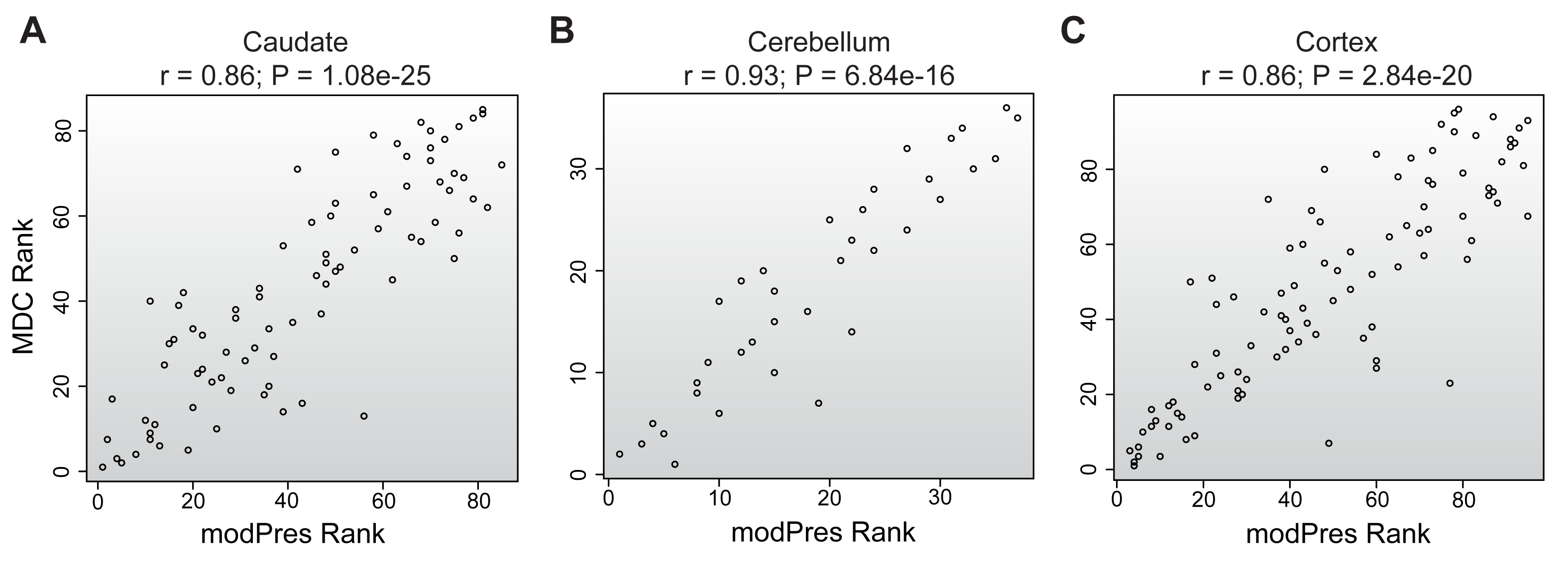

Supplement: S2 Fig — Assessing the reproducibility of the MDC statistic by comparing it to an independent methodology for assessing differential connectivity (medianRank statistic) in the (A) caudate (ρ = 0.86, P = 1.08 x 10^−25^), (B) cerebellum (ρ = 0.93, P = 6.84 x 10^−16^), and (C) frontal cortex (ρ = 0.86, P = 2.84 x 10^−20^). (TIF) [file pgen.1006137.s002.tif]

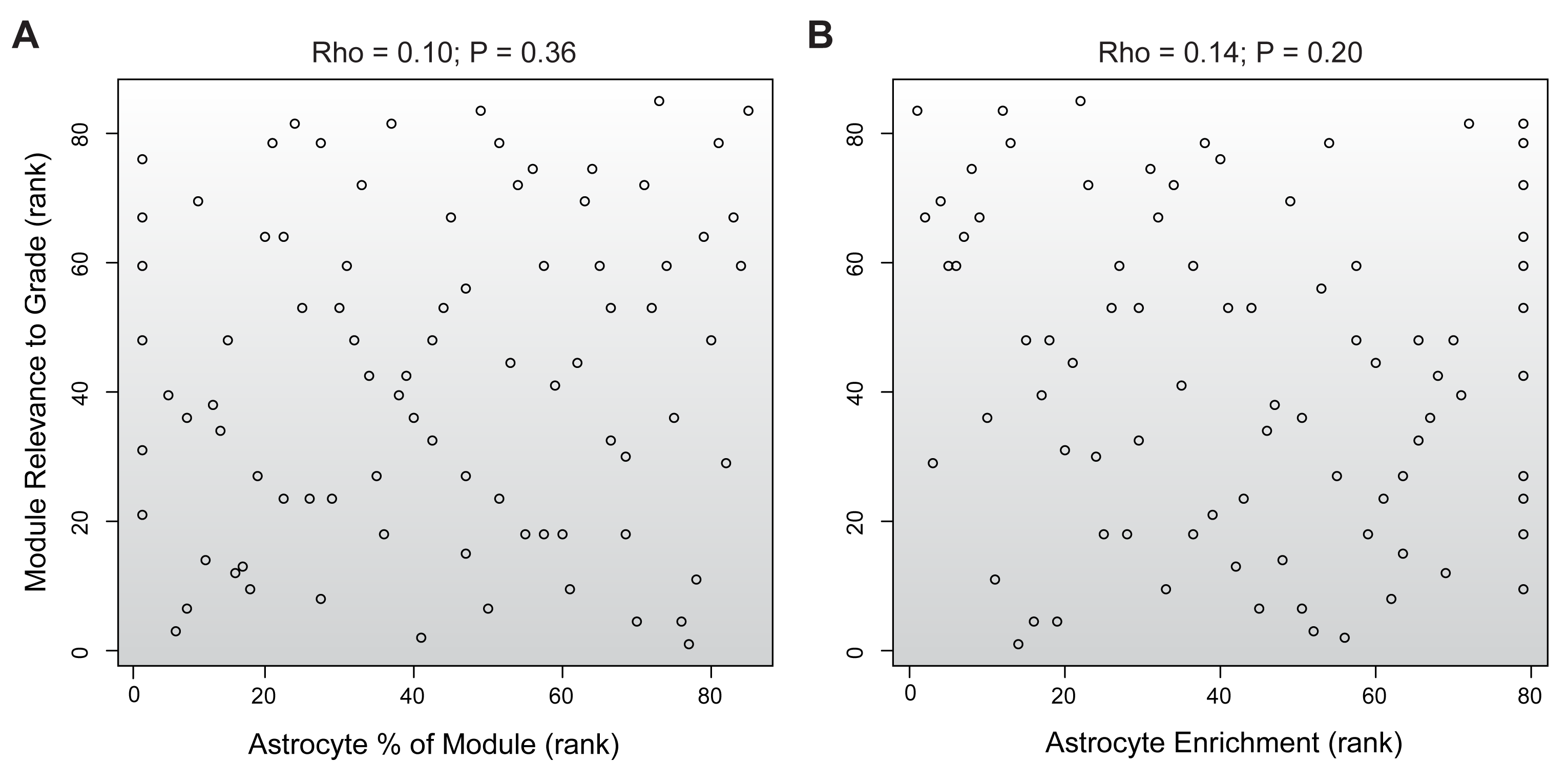

Supplement: S3 Fig — Correlating each module’s association to pathological grade with (A) its enrichment for astrocyte gene signatures and (B) its percentage of astrocyte genes. Pathological grade is not associated to the astrocyte transcriptional profile of modules, suggesting module enrichment is not an artifact of astrocytosis. (TIF) [file pgen.1006137.s003.tif]

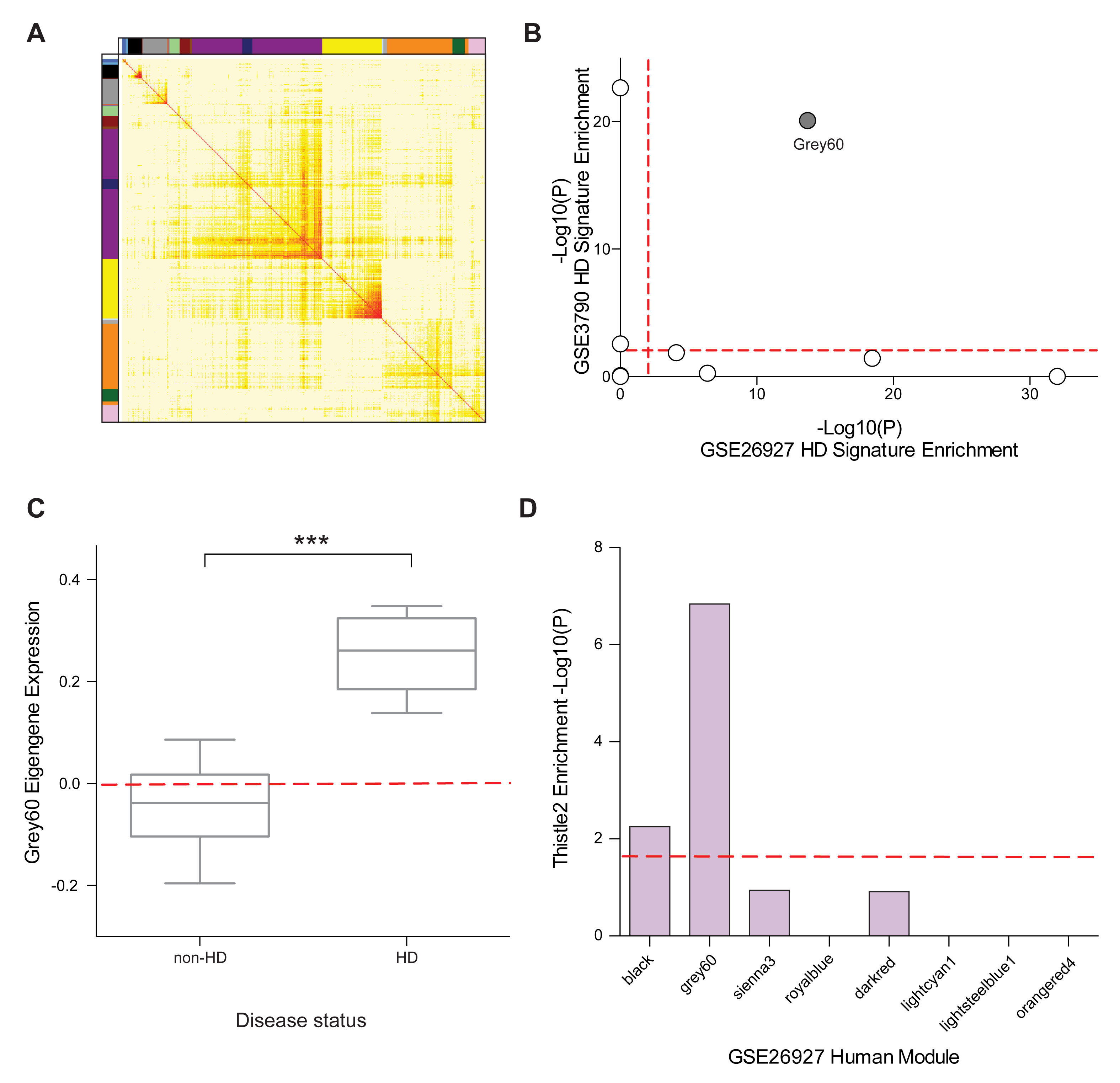

Supplement: S4 Fig — (A) Weighted gene coexpression analysis was performed in an independent validation cohort (N = 62) (GSE26297). In this cohort, multiple brain regions were collected from multiple neuropsychiatric diseases, including the caudate from HD patients. (B) Enrichment for HD-relevant differential expression signatures revealed that Grey60 was most relevant to HD (P < 0.05, two-sided, Bonferroni corrected). (C) Grey60 was also associated to HD status in the caudate (***: P = 3.9e-05, Kruskal-Wallis, Bonferroni corrected, df = 2–1 = 1). (d) Grey60 from our replication cohort (GSE26297) most significantly overlapped with our Thistle2 module from our original cohort (GSE3790). These results show that the caudate astrocyte network is coexpressed in an independent cohort and is most relevant to HD. (TIF) [file pgen.1006137.s004.tif]

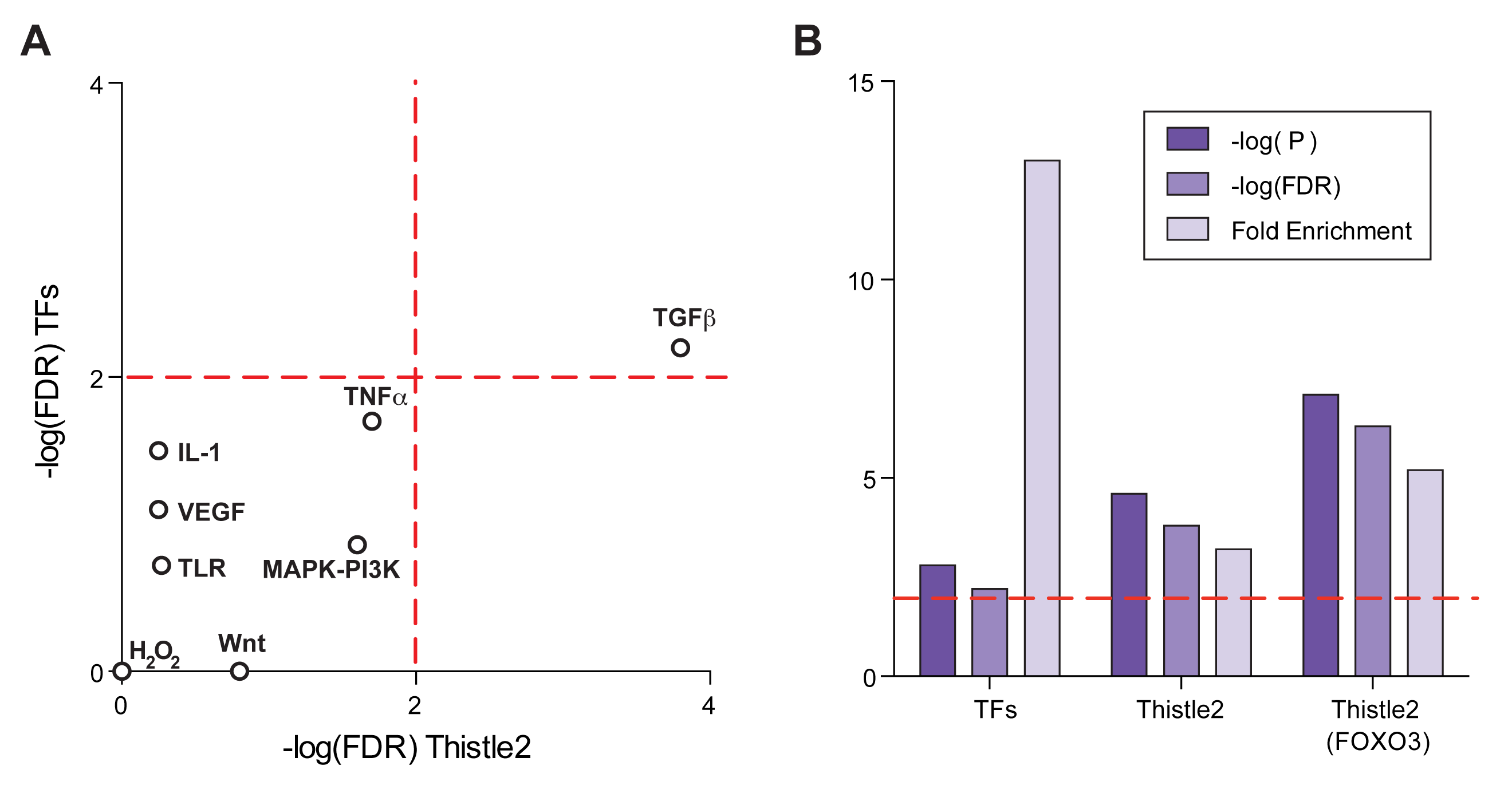

Supplement: S5 Fig — (A) Enrichment of pathway perturbation signatures (Materials and Methods) with Thistle2 and its predicted TFs. Significance threshold (red line): FDR = 0.01. (B) TGFβ pathway enrichment for TFs, Thistle2, and FOXO3-dependent Thistle2. Significance threshold (red line): P = 0.01, FDR = 0.01, Fold enrichment = 2. (TIF) [file pgen.1006137.s005.tif]

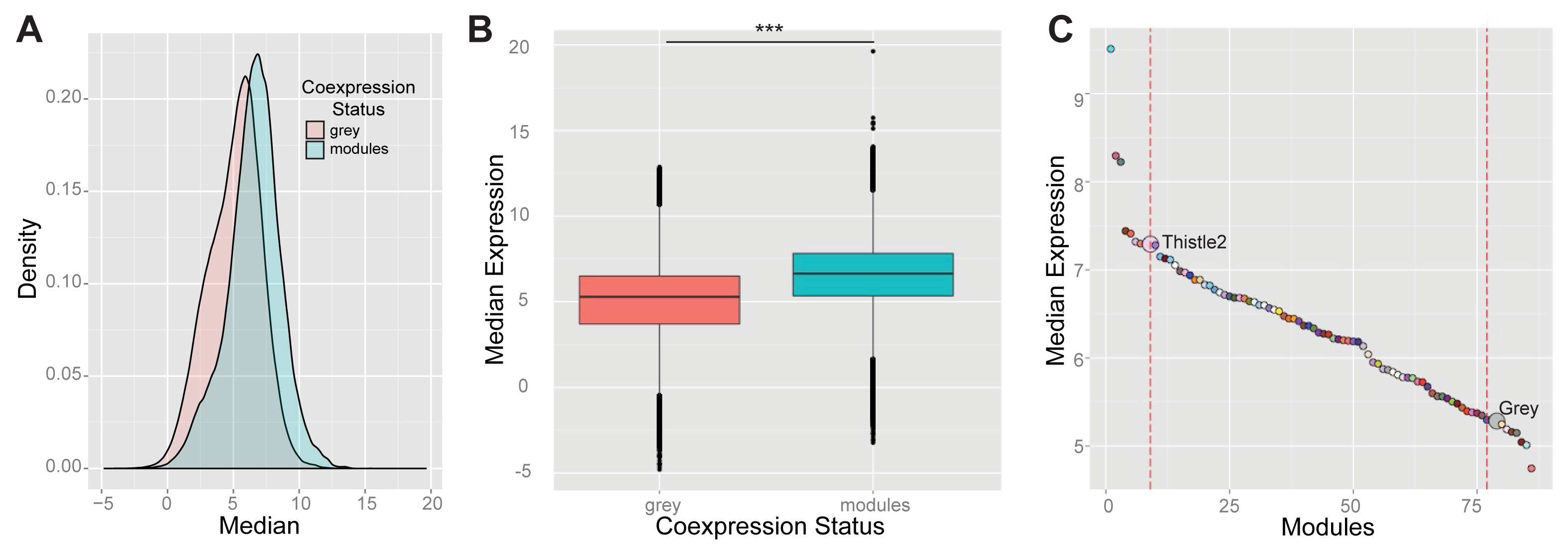

Supplement: S6 Fig — (A,B) Comparison of median expression between genes that cluster in modules and those that do not (grey) (***: Kolmogorov-Smirnov P < 2.2 x 10^−16^). (c) Comparing median expression of genes in each module. Left dotted red line represents the top decile, and the right dotted red line represents the bottom decile. The grey module falls within the bottom decile, as expected, while Thistle2 falls within the top decile. (TIF) [file pgen.1006137.s006.tif]
